# Supplementary figures and images for: Identification of a microbial sub-community from the feral chicken gut that reduces Salmonella colonization and improves gut health in a gnotobiotic chicken model
Source: Microbiol Spectr. 2024 Feb 5;12(3):e01621-23. doi: 10.1128/spectrum.01621-23 (PMC10913435; doi:10.1128/spectrum.01621-23)

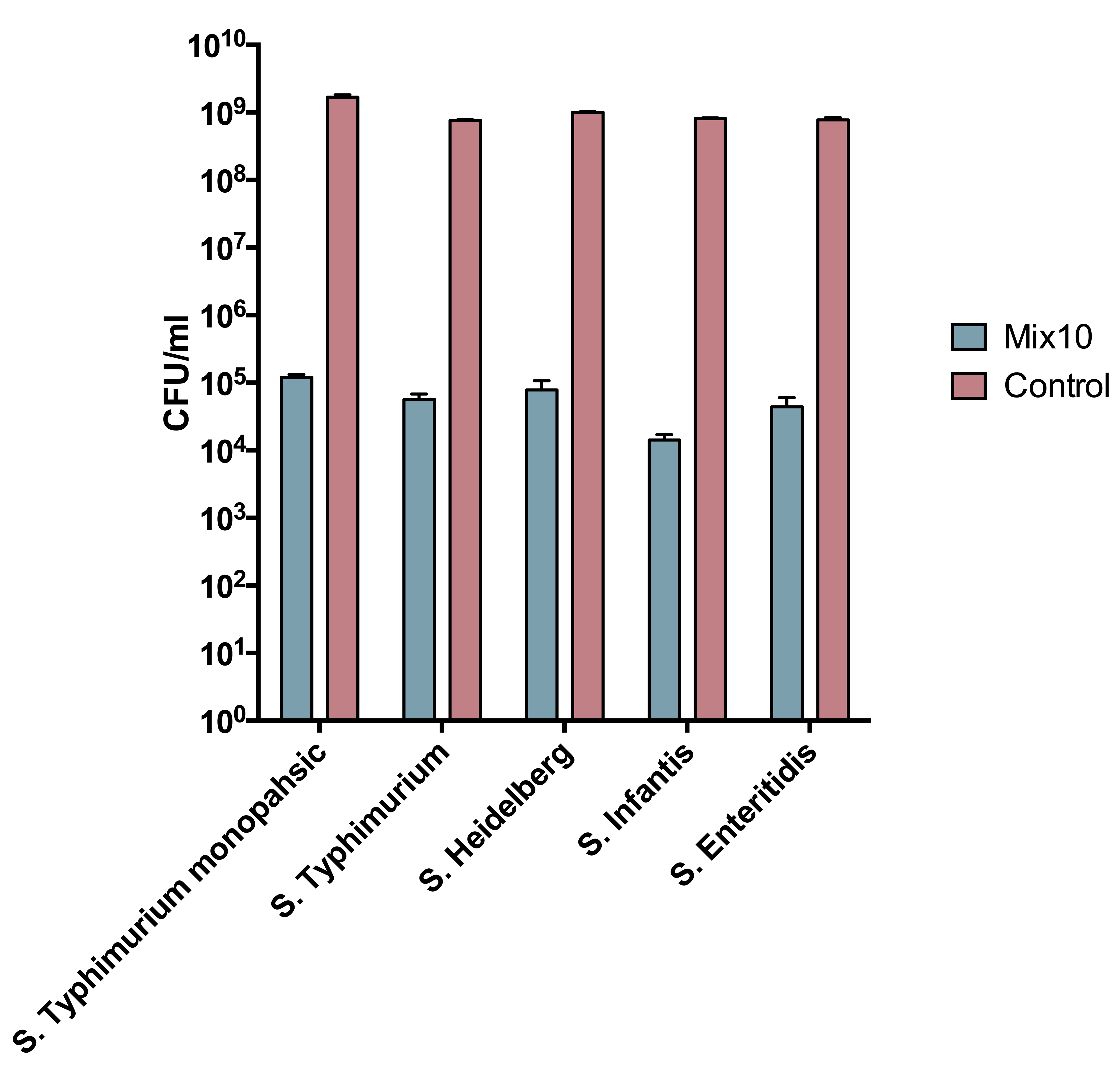

Supplement: Figure S1 — Mix10 inhibition of Salmonella serovars frequently found in poultry. [file spectrum.01621-23-s0001.tiff]

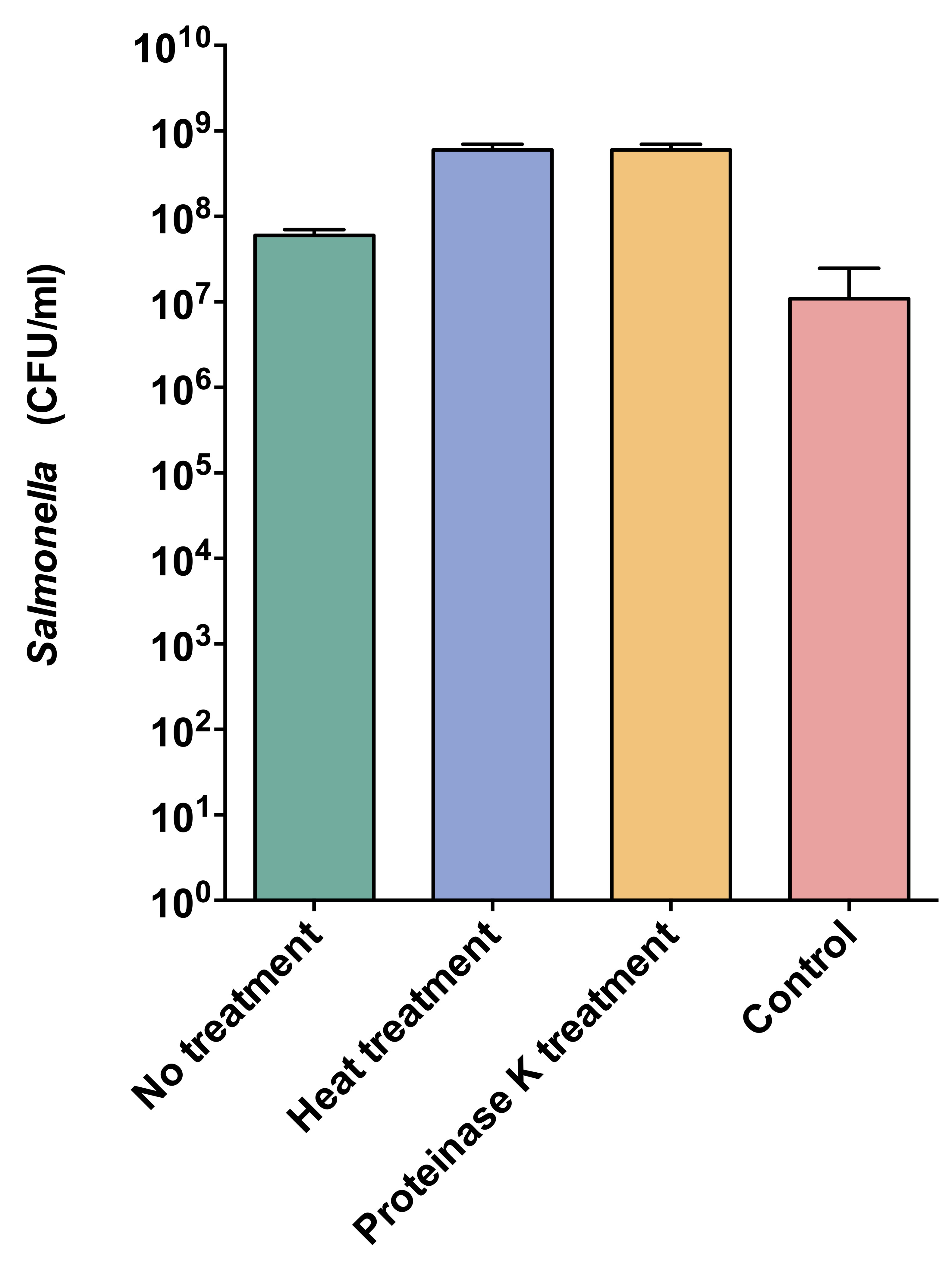

Supplement: Figure S2 — Inhibitory effect of Mix10 cell-free supernatant on S. Typhimurium. [file spectrum.01621-23-s0002.tiff]
