# Supplementary material for: Identification of a microbial sub-community from the feral chicken gut that reduces Salmonella colonization and improves gut health in a gnotobiotic chicken model
Source: Microbiol Spectr. 2024 Feb 5;12(3):e01621-23. doi: 10.1128/spectrum.01621-23 (PMC10913435; doi:10.1128/spectrum.01621-23)
Supplement: Legends — for supplemental figures. [file spectrum.01621-23-s0003.docx]

**Supplementary Figures**

**FIG S1. Mix10 inhibition of Salmonella serovars frequently found in poultry.** Inhibitory capacity of Mix10 against serovars *S*. Typhimurium, *S*. Heidelberg, *S*. Infantis and *S*. Enteritidis were determined using the co-culture assay. The bars show CFU/ml of serotypes of *Salmonella* after 24 h of co-culture with Mix10 (blue bars) and *Salmonella* monoculture (pink bars).

**FIG S2. Inhibitory effect of Mix10 cell-free supernatant on *S*. Typhimurium.** To determine the inhibitory mechanism of Mix10 against Salmonella, a cell-free supernatant co-culture assay was performed. Cell free supernatant of Mix10 was prepared by centrifugation of Mix10 culture at 3,000 rpm for 1 hour. The supernatant was then filtered through 0.4 μm filter. The purified supernatant was adjusted pH to 6.5-6.8 using NaOH and HCl. The supernatant was divided into 3 fractions; no treatment, heat treatment heated at 100^o^C for 1 hour, and 50 μg/ml of proteinase K for 1 hour at 37^o^C. After 24 hours of incubation with cell free supernatant, *Salmonella* CFU was enumerated as described previously.
